# Vertebrobasilar artery cooling infusion in acute ischemic stroke for posterior circulation following thrombectomy: Rationale, design and protocol for a prospective randomized controlled trial

**DOI:** 10.3389/fnins.2023.1149767

**Published:** 2023-04-11

**Authors:** Zhe Cheng, Yuchuan Ding, Gary B. Rajah, Jie Gao, Fenghai Li, Linlin Ma, Xiaokun Geng

**Affiliations:** ^1^Department of Neurology and Stroke Intervention and Translational Center (SITC), Luhe Hospital, Capital Medical University, Beijing, China; ^2^Department of Neurosurgery, Wayne State University School of Medicine, Detroit, MI, United States; ^3^Department of Neurosurgery, Munson Healthcare, Traverse City, MI, United States; ^4^Luhe Institute of Neuroscience, Capital Medical University, Beijing, China

**Keywords:** selective hypothermia, neuroprotection, large vessel occlusion, posterior circulation, endovascular therapy

## Abstract

**Background:**

Although endovascular mechanical thrombectomy demonstrates clinical efficacy in posterior circulation acute ischemic stroke (AIS), only one third of these patients attain functional independence with a third of patients’ expiring despite vascular recanalization. Neuroprotection strategies, such as therapeutic hypothermia (TH) have been considered a promising adjunctive treatment in AIS. We propose the following rationale, design and protocol for a prospective randomized controlled trial (RCT) aimed to determine whether Vertebrobasilar Artery Cooling Infusion (VACI) improves functional outcomes in posterior circulation AIS patients post mechanical thrombectomy.

**Methods:**

Subjects in the study will be assigned randomly to either the cooling infusion or the control group in a 1:1 ratio (*n* = 40). Patients allocated to the cooling infusion group will receive 300 ml cool saline at 4C through the catheter (30 ml/min) into vertebral artery after thrombectomy. The control group will receive the same volume of 37C saline. All patients enrolled will receive standard care according to current guidelines for stroke management. The primary outcome is symptomatic intracranial hemorrhage (ICH), whereas the secondary outcomes include functional outcome score, infarction volume, mortality, ICH, fatal ICH, cerebral vasospasm, coagulation abnormality, pneumonia and urinary infection.

**Discussions:**

This study will determine the preliminary safety, feasibility, and neuroprotective benefits of VACI in posterior circulation AIS patients with reperfusion therapy. The results of this study may provide evidence for VACI as a new therapy in posterior circulation AIS.

**Clinical Trial Registration:**

www.chictr.org.cn, ChiCTR2200065806, registered on November 15, 2022.

## Introduction

Although mechanical thrombectomy (MT) has improved functional outcomes of patients with acute ischemic stroke (AIS) from anterior circulation large vessel occlusion (LVO), data remain less definitive for posterior circulation LVO (Goyal et al., 2016). BEST and BASICS randomized controlled trials (RCTs) did not show significant efficacy of MT in AIS patients with basilar artery occlusion. Conversely, the recent BAOCHE and ATTENTION study provided high-level evidence-based data on the efficacy of posterior circulation thrombectomy, suggesting some effectiveness of MT for patients with basilar artery occlusion (Jovin et al., 2022; Tao et al., 2022). However, similar to anterior circulation thrombectomy, there remains an apparent discrepancy between the successful recanalization (about 90%) and the ability to achieve functional independence after stroke. Only 46% of patients achieved good functional prognosis (mRS 3) at 90 days with more than a 30% mortality rate (Jovin et al., 2022; Tao et al., 2022). We feel more attention must be given to post reperfusion neuroprotection to improve outcomes. The development of rapid and effective neuroprotective strategies is necessary to supplement endovascular reperfusion. Currently no ideal neuroprotection options exist. In this study, we therefore hypothesized Vertebrobasilar Artery Cooling Infusion (VACI) may benefit AIS patients with posterior circulation LVO after endovascular recanalization.

Therapeutic hypothermia (TH) has been shown to be effective in neonates suffering from hypoxic ischemic encephalopathy and patients suffering from cardiac arrest at the clinical level (Edwards et al., 2010; Choi et al., 2012; Nielsen et al., 2013). In recent years, TH has also been considered a promising neuroprotective treatment after AIS and numerous studies have consistently suggested that TH induces multiple synergistic neuroprotective effects in experimental models (Li et al., 2022). Local surface cooling and systemic hypothermia are commonly used in clinical trials. Studies have shown hypothermia in ischemic stroke to be safely inducible (Choi et al., 2012), although few clinical studies have demonstrated efficacy (Wu et al., 2022). Selective endovascular cooling in ischemic brain is a promising approach for neuroprotection. This modality has the potential not only to achieve more rapid cooling but also to avoid the complications related to systemic TH (Wu et al., 2022). A prospective study of 113 consecutive anterior circulation AIS patients suggested that selective intra-arterial cooling infusion targeted into an ischemic brain tissue after endovascular recanalization appears to be safe and can reduce infarct volume (Wu et al., 2018).

Although TH has been performed on anterior circulation AIS patients in vast majority of clinical trials, few studies have assessed TH on posterior circulation stroke. Moreover, to our knowledge, no intra-arterial and selective hypothermia combined with MT for posterior circulation patients has been reported. In the modern mechanical thrombectomy era, reperfusion can be established in most LVO patients and provides an avenue for intra-arterial hypothermia. In order to improve the current outcomes associated with high disability and mortality rates in posterior circulation AIS, therapeutic hypothermia, as a classical neuroprotective strategy, is worth being investigated after recanalization with MT. Thus, we designed this single-center, prospective study to evaluate the safety, feasibility, and preliminary efficacy of intra-arterial regional cooling infusion, the VACI, in posterior circulation AIS after mechanical thrombectomy.

## Methods/design

This study has been approved by the ethics committee of Beijing Luhe Hospital, Capital Medical University, Beijing, China and registered at www.chictr.org.cn (ChiCTR2200065806) in November 2022.

### Study design

This trial is a single-center, prospective, randomized controlled study that aim to evaluate the preliminary safety, feasibility, and efficacy of intra-vertebrobasilar regional hypothermia after MT in posterior circulation AIS patients within 8 h of symptom onset. A physician unaffiliated with the study will monitor the health and safety status of the participants.

### Participants and screening

**Figure 1:**
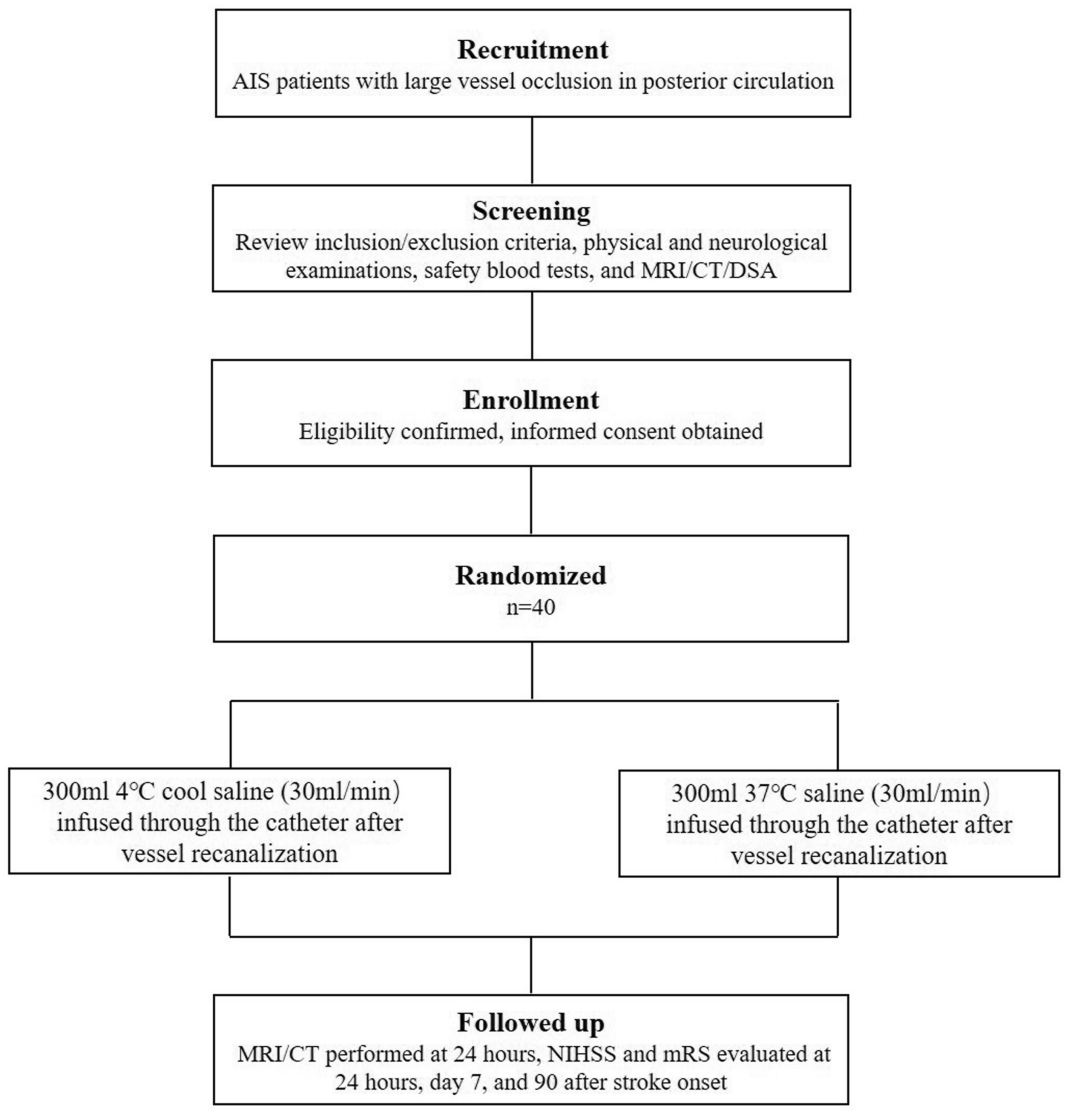
Flowchart of the study.

Eligible participants will be enrolled consecutively after endovascular therapy for acute posterior circulation LVO from stroke center in the Beijing Luhe Hospital, Capital Medical University. Informed consent will be obtained from each participant or an appointed legal representative if the participant lacking decision-making capacity. The flow chart of screening and enrolling see Figure 1.

The inclusion criteria include: (1) age between 18 and 80 years, (2) clinical diagnosis of AIS, (3) posterior circulation LVO confirmed by CTA, MRA or DSA (defined as V4 segment of vertebral artery, basilar artery and posterior cerebral artery), (4) <8 h from onset of symptom to femoral artery puncture, (5) baseline NIHSS (National Institute of Health stroke scale) score ≥8.

The exclusion criteria include: (1) pre-stroke modified Rankin Scale (mRS) >2, (2) evidence suggesting spontaneous vessel recanalization, (3) no vessel recanalization (TICI score ≤1) achieved, (4) rapid neurological recovery after vessel recanalization (NIHSS <6), (5) brain images that indicate large area of bilateral cerebellar ischemia or an bilateral extended brainstem ischemia, (6) severe arterial blood pressure >185/110 mmHg, (7) laboratory studies with evidence of coagulation disorders, (8) blood glucose <2.7 or >22.2 mmol/l, (9) severe cardiac dysfunction, (10) pregnancy, (11) no informed consent able to be obtained, (12) enrolled in other clinical studies within 3 months.

### Randomization and blindness

The eligible patients will be enrolled and randomly allocated to the two groups in a 1:1 ratio (*n* = 40) using a computer-generated randomization method. With the use of opaque envelops, all researchers including interventionalists, outcome assessors, and investigators responsible for data collection and analysis will be blinded to the study.

### Interventions

**Figure 2:**
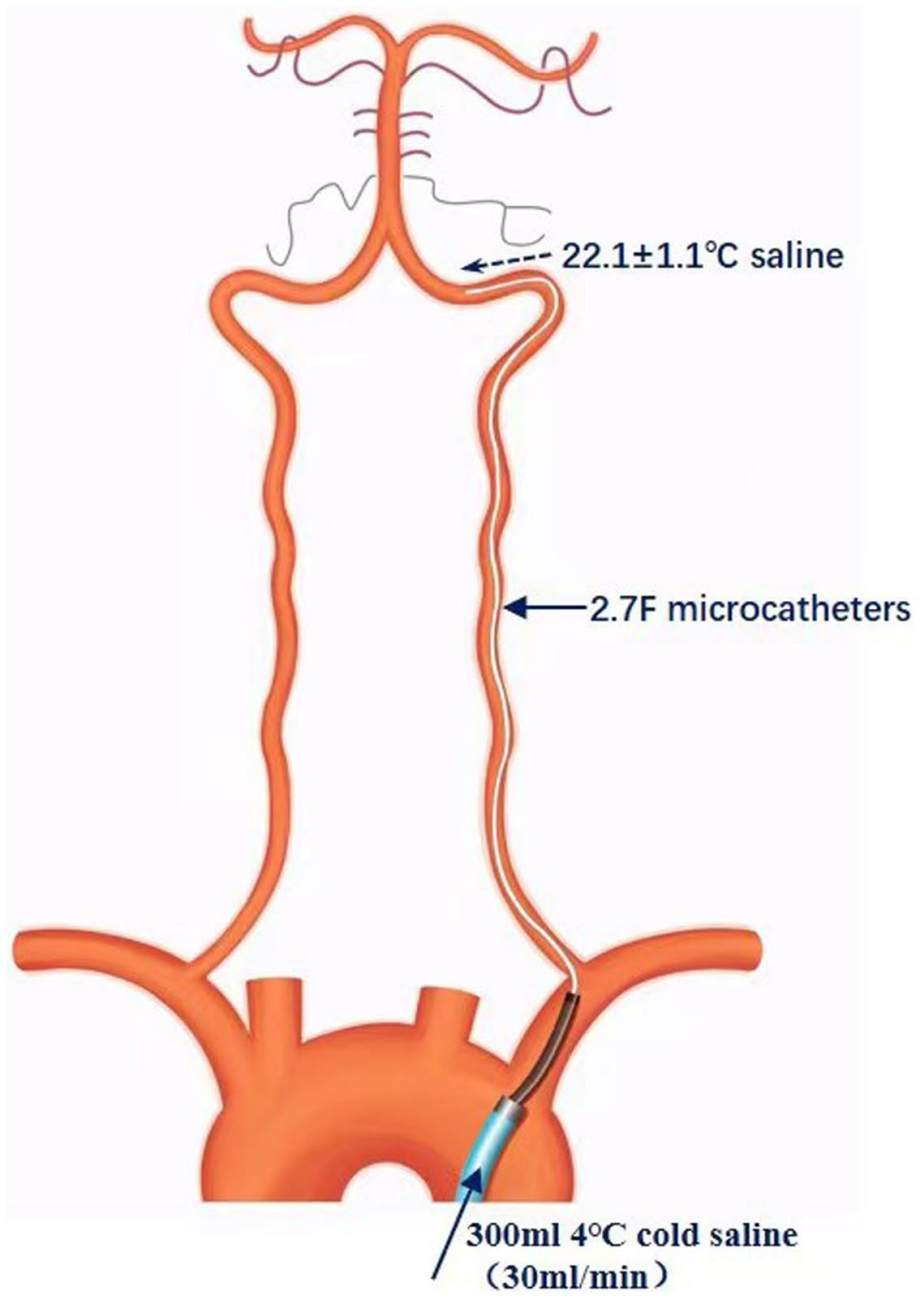
Sketch map of the VACI procedure.

All endovascular procedures were performed under local anesthesia with/without sedative agents. The eligible patients will be randomly allocated to the cooling infusion or control group. All patients within the two groups will receive standard care according to current guidelines, including antiplatelet or anticoagulation treatment, hyperglycemia and hyperlipidemia management, antihypertensive drugs, and treatment of complication. In cooling infusion group, 300 ml 4C cool saline (30 ml/min) will be given in the vertebral artery through a catheter after vessel recanalization as soon as possible (Figure 2). The control group will receive 300 ml 37C saline (30 ml/min) infused into vertebral artery through a catheter. An in-vitro study has demonstrated, 4C cold saline infused at a rate of 30 ml/min through a microcatheters (Excelsior XT-27, Stryker, 2.7F) submerged in a constant temperature bath at 37C (simulating human blood temperature) resulted in the 4C saline that exited from the catheter tip being at 22.1 ± 1.1C (*n* = 6). This would represent the temperature the patient would experience intracranially.

### Outcomes and assessment procedures

The demographic information, risk factors for stroke, comorbidities, occlusion site, procedure of MT, blood pressure, heart rate, finger oxygen saturation, rectal temperature, biochemical indexes before, during, and after VACI will be recorded. NIHSS scores at admission will be determined and collected for further analysis. NIHSS and mRS score will also be evaluated at 24 h, 7 and 90 days after stroke onset. The mRS is a graded interval scale (range, 0 point indicating no symptoms to 6 point indicating death) for the evaluation of neurological functional disability (Hong and Saver, 2009), which will be assessed by trained neurologist blinded to the study. Computerized tomography (CT) will be performed at 24 h after mechanical thrombectomy or whenever any sign of neurological deterioration. Magnetic resonance imaging (MRI) will be performed in a week to evaluate infarction area. Infarct volume will be measured on a Siemens Syngo. *Via* workstation with diffusion-weighted MRI according the method in our previous study (Cheng et al., 2021). Degree of vascular recanalization will be assessed by the modified Thrombolysis in Cerebral Infarction (mTICI) scale (Tung et al., 2017), which will be evaluated by 2 neuroradiologists who are blinded to the assignment of treatment-group.

#### Primary outcomes

The primary outcome of the trial is the incidence of symptomatic intracranial hemorrhage (ICH). ICH is classified into 5 categories according to the European Cooperative Acute Stroke Study II (ECASS 2) (Hacke et al., 1998). Symptomatic ICH (sICH) was defined as parenchymal hemorrhage type I or II diagnosed according to ECASS 2 with an increase in the NIHSS score more than 4-point.

#### Secondary efficacy outcomes

*We will determine*: (1) mRS score at 90 days, (2) favorable prognosis at 90 days (as determined by mRS scores of 0 to 3), (3) mRS scores of 0 to 2 at 90 days, (4) NIHSS scores at 24 h, 7 days, and 90 days, and (5) final infarct volumes.

*Secondary safety outcomes*: Include (1) mortality (mRS score 6), (2) ICH and fatal ICH, and (3) cerebral vasospasm, coagulation abnormality, pneumonia and urinary infection.

### Estimation of sample size

VACI techniques will be the first attempt to our knowledge to address the effect of intra-arterial regional cooling in the posterior circulation. This study will examine the study design, methods, procedures, inclusion criteria, and operational strategies. The results will be used to estimate the sample size and conduct a power analysis for a phase-2 trial. According to previous study in determining sample size for pilot studies (Hertzog, 2008), 10–20 participants enrolled in each group may be sufficient to assess the preliminary trend of safety and feasibility of intra-arterial regional cooling infusion. Therefore, a similar number of 40 participants will be enrolled in this pilot study.

### Statistical analyses

In this study, the intention-to-treat (ITT) principle will be applied to examine end-point events. If the results suggest no difference in the incidence of adverse events found between two study groups and a potential trend of improving functional outcome in cooling infusion group are seen, we will move forward with a phase-2 trial. Before any statistical analysis, the Kolmogorov–Smirnov normality test and equal variance test will be first performed. For normally distributed continuous variables, a two-sided *t*-test will be performed on independent samples to detect the differences between two groups. For continuous variables lacking a normal distribution, a Mann–Whitney U test will be applied. Chi-square or Fisher exact tests will be performed when appropriate for categorical variables. *p* < 0.05 will be considered statistically significant. Statistical analysis will be performed on SPSS 22.0.

## Discussion

At present, the most effective treatment for AIS patients with LVO is revascularization with mechanical thrombectomy. However, the clinical efficacy of thrombectomy therapy is limited by narrow time window, perioperative complications, as well as ischemia and reperfusion injuries such as cerebral edema and intracranial hemorrhage (Siegler and Martin-Schild, 2011; Dong et al., 2017; Nogueira et al., 2018; Ping et al., 2021). In the context of an aging population and rising ischemic stroke events, a concurrent demand to develop neuroprotective therapies is increasing significantly (Wu et al., 2019). Particularly, in posterior circulation AIS, only a third of recanalized patients after MT achieve functional independence, with more than a 30% mortality rate. In order to improve the functional prognosis, developing fast and effective neuroprotective strategies is highly desirable in posterior circulation stroke.

### Beneficial effect of VACI as neuroprotectant

TH is currently recognized as a promising neuroprotective strategy which could exert neuroprotection with action on a broad range of pathological pathways, including the regulation of cerebral blood flow, brain metabolism, microglial activation, inflammation, apoptosis, and neurotrophic factors (Duan et al., 2020; Han et al., 2021; Ma et al., 2021; You et al., 2022). However, clinical application of hypothermia has many limitations. The efficiency of local surface physical cooling is poor to achieve the reduction of intracranial core temperature (Yin et al., 2019). Systemic hypothermia, induced by various vein cooling devices or *via* medication, was feasible in patients with severe AIS, but showed a poor improvement/neuroprotectant effect (Georgiadis et al., 2001; Hemmen et al., 2010; Lyden et al., 2016). The application of hypothermia therapy after stroke has been greatly limited because of the delayed onset of cerebral hypothermia, long hypothermia times, severe complications, as well as great bedside medical and nursing efforts. Intra-arterial cooling infusion which directly targets the ischemic brain tissue thereby would negate the requirement of whole body temperature reduction and the associated side effects and may immediately achieve the targeted temperature within the brain (Konstas et al., 2007; Neimark et al., 2013). Moreover, this unique endovascular technique was able to “flush” the microvasculature of the ischemic territory at risk (Siegler and Martin-Schild, 2011; Savitz et al., 2017; Shimizu et al., 2020), reducing the tissue damage caused by accumulation of inflammatory cells, overproduction of oxygen free radicals and microvascular dysfunction during reperfusion. Depending on removal of those biochemical byproducts and toxic mediators, VACI could greatly minimize reperfusion injury in the ischemic territory. Thus, our VACI model may maximize benefits and minimize side effects related to hypothermia, which is may be promising hypothermia protection strategy for AIS patient.

### Combination of reperfusion therapy and VACI

In recent times, the community of stroke investigators has explored several novel strategies for their potential neuroprotective properties in ischemic stroke. However, most neuro-protectants have only demonstrated reliable efficacy in animal stroke models without successful clinical translation to AIS patients (Babadjouni et al., 2017; Savitz et al., 2017; Ren et al., 2020; Shimizu et al., 2020; Zhao et al., 2020; Ping et al., 2021). Combining such a neuroprotection strategy with reperfusion therapy is considered to be an important impending stage in the development of AIS therapy (Savitz et al., 2017). The recent advances in MT for ischemic stroke have led to high rates of vessel recanalization with new opportunities to successfully apply adjunct neuroprotective treatment to AIS patients after reperfusion (Babadjouni et al., 2017). As a neuroprotective strategy, application of VACI with MT would be effective to improve functional prognosis of stroke patients beyond the levels achieved by current therapies.

### Regional cerebral hypothermia induced by VACI

In the study, 300 ml 4C cold saline will be infused into the ischemic territory through the catheter at a rate of 30 ml/min by which the temperature of cold saline at the outflow end is about 22C. Given a concern about invasive monitor brain injury, and patient compliance, we did not plan to specifically monitor brain temperature with an invasive monitor. However, previous studies have demonstrated an objective relationship between regional cooling and lower target brain temperatures. For example, intracarotid infusion of cold saline (4–10C) at rate of 33 ml/min can led to a rapid temperature reduction in the jugular venous bulb by 0.84 ± 0.1C within 8.2 min in patients undergoing cerebral angiography (Choi et al., 2010). On the basis of the above study and same cooling method, a three-dimensional human brain model was designed in another study, which inferred that the temperature of the ipsilateral anterior circulation cerebral territory decreased by approximately 2C within 10 min (Neimark et al., 2013). In another theoretical model, intra-carotid cold saline infusion at rate of 30 ml/min appears to be sufficient to induce moderate hypothermia (33–34C) within 10 min in the ipsilateral cerebral hemisphere (Konstas et al., 2007). Furthermore, intra-arterial cooling infusion is 18–42 times faster than the 3–7 h needed for whole body cooling by noninvasive methods to achieve rapid cerebral hypothermia (Kammersgaard et al., 2000; Leker and Ovadia, 2002). Thus, we speculate that the VACI model used in our study could rapidly and effectively reduce the temperature of ischemic brain tissue in posterior circulation territory.

### Study limitations

This proposed study has several limitations. First, it is a single-center trial with a small sample size, which may influence the generalizability of the conclusions and may potentially invite confounding results to the study. Second, the target volume and velocity of infusion solution needs to be optimized.

The trial is designed to identify the safety, feasibility, and, secondarily, the possible efficacy of VACI combined with MT in posterior circulation AIS patients. The preliminary results will provide the parameters for future clinical trials, purely focusing on efficacy.

## Ethics statement

This study has been approved by the ethics committee of Beijing Luhe Hospital, Capital Medical University, Beijing, China. Study safety and quality control meeting will be held twice a year and on an as needed basis by the Good Clinical Practice (GCP) committee of Beijing Luhe Hospital. The safety of participants in the study will be continuously monitored by the Ethics Committee.

## Author contributions

YD, XG, and ZC designed the study. ZC, JG, FL, and LM prepared the manuscript. YD and GR revised the manuscript. All authors contributed to the article and approved the submitted version.

## Funding

This study was supported partially by the Capital Clinical Special Projects (Z221100007422085) and National Natural Science Foundation of China (8207091513). The funding body had no role in the design of the study and collection, analysis, and interpretation of data and in writing the manuscript.

## Conflict of interest

The authors declare that the research was conducted in the absence of any commercial or financial relationships that could be construed as a potential conflict of interest.

## Publisher’s note

All claims expressed in this article are solely those of the authors and do not necessarily represent those of their affiliated organizations, or those of the publisher, the editors and the reviewers. Any product that may be evaluated in this article, or claim that may be made by its manufacturer, is not guaranteed or endorsed by the publisher.
